# Validation of the Arabic Maternal Breastfeeding Evaluation Scale (MBFES-A) among Lebanese women

**DOI:** 10.1186/s13006-021-00409-w

**Published:** 2021-08-21

**Authors:** Mona Nabulsi, Hanan Smaili, Hani Tamim, Marya Wahidi, Carine El-Jamal

**Affiliations:** 1 Department of Pediatrics and Adolescent Medicine, American University of Beirut, Beirut, Lebanon; 2 Clinical Research Institute, Biostatistics Unit, American University of Beirut, Beirut, Lebanon; 3 Faculty of Arts and Sciences, Medical Research Volunteer Program (MRVP), American University of Beirut, Beirut, Lebanon

**Keywords:** Breastfeeding evaluation, Instrument validation, Maternal satisfaction, Arab countries, Exclusive breastfeeding, Infant satisfaction, Infant growth

## Abstract

**Background:**

Maternal satisfaction with the breastfeeding experience is an important determinant of breastfeeding success. There is currently no valid tool to measure perceived maternal satisfaction with breastfeeding in the Arab context.

**Methods:**

This cohort study tested the Maternal Breastfeeding Evaluation Scale (MBFES) on 450 healthy Lebanese mothers for internal consistency reliability and construct validity. Participants were recruited between April 2018 and February 2020.

**Results:**

The Cronbach’s alpha reliability coefficient of the Arabic MBFES (MBFES-A) was 0.87. Exploratory factor analysis revealed that it has three components: Infant Satisfaction/Growth, Maternal Enjoyment/Role Attainment, and Lifestyle/Body Image with reliability coefficients of 0.88, 0.87, and 0.68, respectively. Four items were deleted because of low factor loadings and three items were relocated to the Infant Satisfaction/Growth subscale based on their factor loadings. Participants who were exclusively breastfeeding at one and/or 3 months had higher mean MBFES-A total and Infant Satisfaction/Growth and Maternal Enjoyment/Role Attainment subscale scores than participants who were partially breastfeeding, and significantly higher mean scores than mothers who were not breastfeeding (all *p* values < 0.001), findings that support the scale’s construct validity. Moreover, scores on the Infant Satisfaction/Growth subscale correlated with exclusive breastfeeding at one (*r* = 0.37, *p* < 0.001) and 3 months (*r* = 0.31, *p* < 0.001). The MBFES-A score had positive modest correlations with maternal attitude towards breastfeeding (*r* = 0.30, *p* < 0.001), exclusive breastfeeding at one (*r* = 0.27) and at 3 months (*r* = 0.26, *p* < 0.001 for both), as well as with the longest previous exclusive breastfeeding (*r* = 0.27, *p* < 0.001).

**Conclusions:**

The 26-item MBFES-A is a reliable and valid instrument to use in future breastfeeding research in Middle East North Africa countries. There is a need for replication of our findings in other Arab contexts using new constructs to establish stronger construct validity.

**Supplementary Information:**

The online version contains supplementary material available at 10.1186/s13006-021-00409-w.

## Background

The short- and long-term benefits of breastfeeding for mothers and their children are well-supported by strong evidence. As such, breastfeeding is considered one of the most important public health measures to improve maternal and childhood outcomes [1–7]. However, the practice of breastfeeding remains low worldwide [1], including countries in the Middle East/North Africa (MENA) region [8–11]. Lebanon is a country with low breastfeeding rates. Although a high initiation rate of 96% has been previously reported [12], exclusive breastfeeding prevalence drops to 41.5% at 40 days postpartum [13], down to 12.3–15% in infants below 6 months [13, 14].

The low rates of breastfeeding in the MENA region call for further research to support the promotion of breastfeeding, and the implementation of effective interventions that can positively impact the existing low breastfeeding rates. One of the important barriers to breastfeeding research in the MENA region is the scarcity of validated instruments that can measure the different aspects relevant to breastfeeding practice. Perceived maternal satisfaction with the breastfeeding experience for example may be an important determinant of breastfeeding success. There is only one validated English instrument, the Maternal Breastfeeding Evaluation Scale (MBFES) designed to measure maternal perceived overall quality of a mother’s breastfeeding experience. The MBFES was developed in the United States by Leff, Jefferis, and Gagne [15], and validated on a sample of American mothers by Riordan, Woodley, and Heaton [16]. It has been used in studies from several English-speaking countries [17–23], and validated in Japanese [24, 25] and Brazilian contexts [26], but to date, there is no validated version in Arabic. This study aimed at adapting and validating the MBFES in a cohort of healthy Lebanese mothers, as well as investigating the association between maternal satisfaction with breastfeeding and actual breastfeeding at one and 3 months postpartum. The availability of a validated tool to measure maternal satisfaction with breastfeeding experience is essential for Arab investigators conducting breastfeeding research in the MENA region.

## Methods

### Design and setting

This is a cohort instrument validation study that was conducted in a tertiary care center in Beirut Lebanon. The center is an academic, non-profit, privately funded hospital that serves middle to high income patients, mostly from the capital city Beirut.

### Sample

Healthy Lebanese women who delivered a healthy singleton newborn were recruited as they presented to the postpartum ward on their first day after delivery. Inclusion criteria were intention to breastfeed after delivery and being able to read and write in Arabic. Women were excluded if they had a chronic medical condition, did not want to breastfeed, had a twin pregnancy, delivered prematurely before 37 weeks of gestation, or if the infant was admitted to the neonatal intensive care unit.

### Original instrument

The MBFES questionnaire measures maternal perceived overall quality of the breastfeeding experience [15, 16]. It is composed of 30 items that are divided into 3 categories: Maternal Enjoyment/Role Attainment (14 items), Infant Satisfaction/Growth (8 items), and Lifestyle/Body Image (8 items). The MBFES has a Cronbach’s alpha of 0.93, with reliability coefficients of 0.93 for the Maternal Enjoyment/Role Attainment, 0.88 for the Infant Satisfaction/Growth, and 0.80 for the Lifestyle/Body Image categories. The questionnaire uses a 5-point Likert-type scale for scoring item responses, ranging from 1 (*strong disagreement*) to 5 (*strong agreement*). Of the 30 items, 19 are positively worded statements about breastfeeding and thus are positively scored, while 11 items are negative statements that are scored in reverse. The sum of all MBFES scores results in a minimum of 30 and a maximum of 150 points, with higher scores indicating more satisfaction with the breastfeeding experience.

### Cross-cultural adaptation

One of the authors translated the MBFES to Arabic. It was then back translated to English by an independent bilingual translator who was unaware of the original wording of the questionnaire. The back translated English version was compared to the original English MBFES for accuracy and was found to be similar. The Arabic MBFES was then piloted on 20 Lebanese mothers to assess its clarity, comprehension, length and cultural acceptability. These women were healthy mothers who had delivered a healthy newborn at the same center, and were presenting for their one-month postpartum check at the Women’s Health Center (*n* = 5), the Obstetric out-patient department (*n* = 5), or to the Pediatric ambulatory clinic for the infant’s one-month well child check (*n* = 10). All participants reported that the piloted Arabic MBFES was clear, easy to understand and of adequate length. As for cultural acceptability, all except one participant approved the scale’s content. This participant commented on item 27 (*Breastfeeding made me feel like a cow*) as being culturally inappropriate. Since the piloted version was approved by almost all participants, no changes were made to the translated questionnaire including item 27.

### Data collection

Participants were recruited by trained research assistants who interviewed them in the privacy of their postpartum rooms. After explaining the study purpose and procedures, and obtaining the written informed consent, the assistants administered a standardized questionnaire to collect the following socio-demographic data: age, highest education attained by the participant, employment status, household monthly income, gestational age, number of living children, number of breastfed children, having support at home, mode of delivery, newborn’s gender, newborn’s birth weight, and longest durations of previous exclusive breastfeeding and any breastfeeding (in multiparous women). The longest duration of previous exclusive breastfeeding was defined as the longest period (in months) during which a multiparous participant breastfed a son/daughter. Participants were also administered the validated Arabic Breastfeeding Knowledge questionnaire (BFK-A) to assess maternal breastfeeding knowledge [27], and the validated Arabic Iowa Infant Feeding Attitude Scale (IIFAS-A) [28] to evaluate maternal attitude towards infant feeding methods.

The research assistants contacted the participants by telephone at two weeks, one month, and three months to collect information on the infants’ feeding method. An infant was on exclusive breastfeeding if he/she was feeding human milk only, with no other food or drink including water, but allowing oral rehydrating solutions, vitamins, minerals, or other medicines when needed [29]. In addition, participants were administered the Arabic MBFES at one month via telephone survey.

### Data analysis

We summarized continuous variables as means and standard deviations (*SD*) or medians and interquartile ranges (*IQR*) as appropriate, and categorical variables as frequencies and proportions. Continuous variables were compared using independent *t* test or ANOVA as needed and categorical variables were compared using *Chi* Square test.

The MBFES responses were scored in accordance with the scoring reported in the original MBFES study [15]. We conducted an Exploratory Factor Analysis (EFA) on the 30-item Arabic MBFES to assess dimensionality and construct validity using Principal Component Analysis (PCA) with varimax rotation. To test the suitability of the PCA method, we ran the Kaiser-Meyer-Olkin (KMO) measure of sample adequacy and Bartlett’s test of sphericity. We checked the scree plot and the Eigenvalues to decide on the number of factors that the items were loading on. Moreover, we assessed the internal consistency reliability of the Arabic MBFES using Cronbach’s alpha coefficient, as well as its item-total statistics (e.g. item-total correlations and scale reliability coefficient if an item was deleted) to decide on the items to be retained. Since the original MBFES had three subscales, we planned to assess the internal consistency reliability of potential subscales of the Arabic MBFES using Cronbach’s alpha coefficient.

To demonstrate construct validity, we hypothesized that: 1) mothers breastfeeding at one and three months would have higher scores on the MBFES scale and its subscales; 2) mothers who breastfed previous infants for longer durations would be more satisfied with their breastfeeding experience; 3) participants with a more positive attitude towards breastfeeding and/or better breastfeeding knowledge would have higher breastfeeding satisfaction scores than participants with negative breastfeeding attitudes or poor knowledge. The scale’s construct validity was thus assessed by comparing the participants’ scores on the Arabic MBFES (overall and subscale scores) to their actual breastfeeding at one and three months. The associations between actual breastfeeding at one month and MBFES total and subscales' scores, as well as between actual breastfeeding at three months and MBFES scores were investigated using Pearson’s correlation coefficient (*r*). Moreover, the association between the MBFES scores and each of the longest duration of previous exclusive breastfeeding, IIFAS-A scores, and BFK-A scores were similarly investigated. All analyses were done using SPSS version 23. Statistical significance was set at a *p* value of < 0.05.

### Ethical considerations

This study was approved by the institutional review board of the American University of Beirut (Protocol PED.MN.16/SBS-2017-0450). All participants provided written informed consent prior to their participation in the study.

## Results

### Characteristics of the sample

Between April 2018 and February 2020, 485 participants were recruited. Of these, 39 participants withdrew or were lost to follow up during the study (24 at 2 weeks, 11 at one month, and 2 at three months). Table 1 details the baseline characteristics of the 485 participants. Slightly more than half of the participants were multiparous (*n* = 267, 55.1%), and 284 (58.6%) participants delivered by normal vaginal delivery. Of the multiparous women, 97 (39.8%) stated that they never breastfed their children.

**Table 1:** Baseline characteristics and breastfeeding outcomes (*N* = 485)

Continuous Variables, normal distribution	Mean (***SD***)
Age (Years)	31.1 (4.9)
Gestation (Completed weeks)	38.4 (1.3)
Infant’s birth weight (Grams)	3265.6 (405.1)
BFK-A score	12.5 (2.0)
IIFAS-A score	67.0 (7.2)
**Continuous Variables, skewed distribution**	**Median (*IQR*)**
Number of children	1 (0, 1)
Range	0–5
†Number of breastfed children	1 (1, 2)
Range	0–5
†Longest duration of previous EBF (months)	3 (0.0, 11.8)
Range	0.0–29.0
†Longest duration of any previous BF (months)	1.0 (0.0–5.0)
Range	0.0–30.0
**Categorical Variables**	***n*(%)**
Primiparous	218 (44.9)
Cesarean delivery	201 (41.4)
Male infant	231 (47.6)
Education	
< University	64 (13.2)
≥ University	421 (86.8)
Employed	293 (60.4%)
Full-time	246 (84.0)
Can leave work to BF	99 (33.8)
Can pump at work	243 (82.9)
‡Monthly income ($)	
≤ 1000	63 (13.2)
1001- < 5000	304 (63.7)
≥ 5000	110 (23.1)
Has support at home	470 (96.9%)
Infant nutrition at 2 weeks	
EBF	248 (53.8)
Mixed feeding	183 (39.7)
Artificial milk	30 (6.5)
Infant nutrition at 1 month	
EBF	216 (48.0)
Mixed feeding	183 (40.7)
Artificial milk	51 (11.3)
Infant nutrition at 3 months	
EBF	172 (38.4)
Mixed feeding	147 (32.8)
Artificial milk	129 (28.8)

EBF = exclusive breastfeeding; BF = breastfeeding; †For multiparous participants; ‡Missing values: longest duration of EBF = 23, longest duration of any BF = 23, monthly income = 8, infant nutrition at 2 weeks = 24, infant nutrition at 1 month = 35, infant nutrition at 3 months = 37

The participants had a mean (*SD*) MBFES score of 111.7 (13.6). Their mean (*SD*) BFK-A score was 12.5 (2.0) with 54% having good or very good breastfeeding knowledge (BFK-A score above the mean). For the IIFAS-A, the mean (*SD*) score was 67.0 (7.2) with 68.4% having a neutral attitude towards breastfeeding (IIFAS-A score between *mean- 1 SD* and *mean + 1 SD),* and 16.1% having a strong attitude towards breastfeeding (IIFAS-A score above *mean + 1 SD*). The rate of exclusive breastfeeding during the study dropped from 53.8% at two weeks to 38.4% at three months postpartum (Table 1).

### Internal consistency reliability

**Fig. 1:**
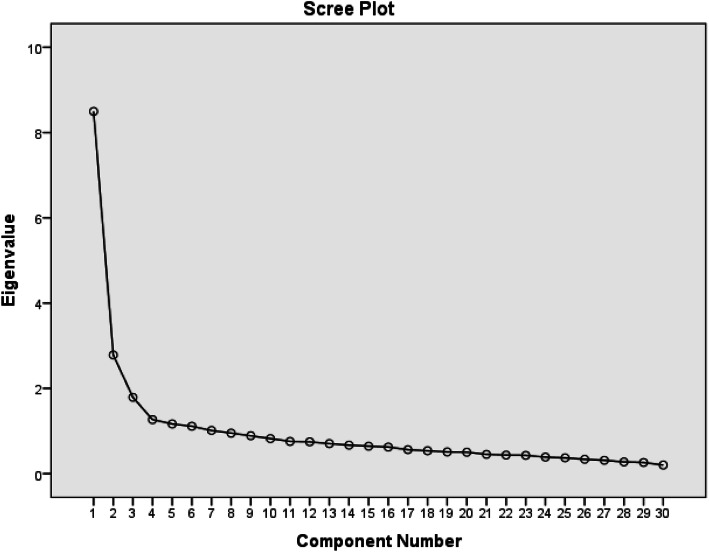
Scree plot of the MBFES-A revealing four points above the curve’s “elbow” (Eigen values above 1)

The Cronbach’s alpha internal consistency reliability of the MBFES was 0.87. Inter-item correlations ranged between − 0.001 and 0.735, with corrected-item total correlations ranging from 0.178 for item 15 to 0.625 for item 12. Cronbach’s alpha if item deleted ranged between 0.86 and 0.88 suggesting that none of the items needed to be re-evaluated or dropped. The KMO measure of sample adequacy was 0.911 (*p* < 0.001), which implies that it was adequate for PCA [30]. The scree plot that was generated from EFA (Fig. 1) suggested that the scale has three or four components with Eigen values above 1. The 4-component factor loadings had a total variance of 47.8% with 28 items. The four components were Maternal Enjoyment/Role Attainment (variance of 28.3%), Infant Satisfaction (9.3%), Lifestyle/Body Image (6.0%) and Infant Growth (4.2%). Items 13 (*While breastfeeding, I felt self-conscious about my body*) and 29 (*Breastfeeding was emotionally draining*) were excluded because their factor loadings were below 0.4 (Table 2). The Cronbach’s alpha reliability coefficient of the 28-item MBFES was 0.88 and those of the four factors were 0.87 for Maternal Enjoyment/Role Attainment, 0.72 for Infant Satisfaction, 0.68 for Lifestyle/Body Image, and 0.60 for Infant Growth.

**Table 2:** Factor loadings (*N* = 450)

Infant satisfaction/growth	Factors		
	1	2	3
-Baby loved to nurse	**0.738**		
-Baby did not relax while nursing	**0.721**		
-Baby wasn’t interested with breastfeeding	**0.700**		
-Baby was an eager breast-feeder	**0.700**		
-Breastfeeding was soothing when my baby was upset and crying	**0.644**		
-While breastfeeding, my baby’s growth was excellent	**0.642**		
-My baby and I worked together to make breastfeeding go smoothly	**0.617**		
-The fact that I could produce the food to feed my own baby was very satisfying	**0.591**		
-Baby gained weight really well with breast milk	**0.576**		
**Maternal enjoyment/Role attainment**			
-Breastfeeding made me feel a more confident mother		**0.716**	
-With breastfeeding I felt a sense of inner contentment		**0.665**	
-I felt extremely close to my baby when I breastfed		**0.655**	
-Breastfeeding made me feel like a good mother		**0.647**	
-Breastfeeding felt wonderful to me		**0.646**	−0.43
-I really enjoyed nursing		**0.627**	−0.44
-Breastfeeding was a very nurturing, maternal experience	0.44	**0.570**	
-Breastfeeding made my baby feel more secure	0.44	**0.539**	
-Breastfeeding was like a high of sorts		**0.541**	−0.47
-Breastfeeding was a special time with my baby		**0.493**	
-It was important to me to be able to nurse		**0.480**	
**Lifestyle/Body image**			
-While breastfeeding, I felt too tied down all the time			**0.727**
-Breastfeeding was physically draining			**0.708**
-I could easily fit my baby’s breastfeeding with my other activities			**0.616**
-It was a burden being my baby’s main source of food			**0.543**
-Breastfeeding made me feel like a cow			**0.420**
-While breastfeeding, I was anxious to have my body back			**0.417**

On the other hand, the 3-component factor loadings had a total variance of 43.6%. Like the original 30-item English MBFES, the components were Infant Satisfaction/Growth, Maternal Enjoyment/Role Attainment, and Lifestyle/Body Image, accounting for 28.3, 9.3, and 6.0% of the total variance, respectively. Items with factor loadings below 0.4 were deleted. These were items 13 and 29 (same items deleted in the 4-component factor loadings), in addition to items 15 (*While breastfeeding, I worried about my baby gaining enough weight*) and 19 (*In the beginning, my baby had trouble breastfeeding*). Excluding these four items reduced the number of items to 26 instead of 30 items. The Cronbach’s alpha reliability coefficient of the 26-item MBFES was 0.89. Reliability coefficients of the subscales were 0.88 for Infant Satisfaction/Growth, 0.87 for Maternal Enjoyment/Role Attainment, and 0.68 for Lifestyle/Body Image. We chose the 26-item scale to be the adapted Arabic MBFES (MBFES-A) since its reliability coefficient and those of its subscales were higher than the 4-component factor scale reliability coefficients. Moreover, the three subscales were more comparable to those of the original English 30-item MBFES. The adapted 26-item MBFES-A is shown in Additional file 1. Three items were relocated from Maternal Enjoyment/Role Attainment to Infant Satisfaction/Growth because they loaded on the latter factor. These items were: a) “*My baby and I worked together to make breastfeeding go smoothly*”, b) “*Breastfeeding was soothing when my baby was upset and crying*”, and c) “*The fact that I could produce the food to feed my own baby was very satisfying*” (Table 2).

### Construct validity

Participants who at 1 month were exclusively breastfeeding had higher mean (*SD*) scores on the 26-item MBFES-A and the Infant Satisfaction/Growth and Maternal Enjoyment/Role Attainment subscales than participants whose infants were on mixed feeding, and significantly higher mean (*SD*) scores than mothers feeding their infants formula milk (Table 3). Similarly, the mean (*SD*) scores on the 26-item MBFES-A and the Infant Satisfaction/Growth and Maternal Enjoyment/Role Attainment subscales at three months were highest in mothers who continued exclusive breastfeeding for three months, followed by mothers whose infants were on mixed feeding, and were lowest in mothers who were feeding their infants formula milk (Table 3). At one month, the Pearson correlations between exclusive breastfeeding and the MBFES-A were as follows: *r* = 0.27 for the overall score, *r* = 0.37 for Infant Satisfaction/Growth, *r* = 0.22 for Maternal Enjoyment/Role Attainment (all *p* values < 0.001), and *r* = 0.01 for Lifestyle/Body Image (*p* = 0.797). At three months, the correlations were *r* = 0.26 for the overall score, *r* = 0.31 for Infant Satisfaction/Growth, *r* = 0.22 for Maternal Enjoyment/Role Attainment (all *p* values < 0.001), and *r* = 0.06 for Lifestyle/Body Image (*p* = 0.248).

**Table 3:** Participants’ scores on the 26-item Arabic MBFES and its subscales by type of infant feeding at 1 and 3 months

Scale/Subscale	EBF *n* = 172 Mean (*SD*)	Mixed Feeding *n* = 146 Mean (*SD*)	Formula milk *n* = 127 Mean (*SD*)	^a^***p*** Value
**At 1 month**				
26-item MBFES-A	108.1 (9.7)	103.8 (12.1)	91.2 (17.8)	< 0.001
Infant Satisfaction/Growth	40.3 (3.6)	37.3 (5.4)	30.9 (7.9)	< 0.001
Maternal Enjoyment/Role Attainment	49.0 (4.7)	47.6 (5.5)	41.9 (8.8)	< 0.001
Lifestyle/Body Image	18.9 (4.5)	18.9 (4.7)	18.5 (5.2)	0.861
**At 3 months**				
26-item MBFES-A	108.8 (9.3)	105.7 (10.6)	97.4 (16.0)	< 0.001
Infant Satisfaction/Growth	40.3 (3.4)	39.0 (4.3)	33.9 (7.4)	< 0.001
Maternal Enjoyment/Role Attainment	49.3 (4.5)	48.3 (4.7)	44.6 (7.7)	< 0.001
Lifestyle/Body Image	19.2 (4.4)	18.4 (4.8)	18.9 (4.8)	0.341

^a^ANOVA, *SD* = standard deviation, MBFES-A = Arabic Maternal Breastfeeding Evaluation Scale, EBF = exclusive breastfeeding

The 26-item MBFES-A score had positive modest correlations with the IIFAS-A score (*r* = 0.30, *p* < 0.001) and the longest duration of previous exclusive breastfeeding (*r* = 0.27, *p* < 0.001) while poor correlations were found with the number of breastfed children (*r* = 0.12, *p* = 0.013), and the BFK-A score (*r* = 0.12, *p* = 0.014). There were no significant associations between the 26-item MBFES-A score and the participant’s age, length of gestation, number of children, infant’s birth weight or gender.

## Discussion

The adapted 26-item MBFES-A is a reliable instrument in our context as it has similar components to the original English MBFES with comparable reliability coefficients [15, 16]. It has four less items than the original English scale because their factor loadings were low. Three of these items (items 13, 15, and 19) were also excluded from the Japanese MBFES [24]. The deletion of these four items did not affect the reliability coefficients of the MBFES-A scale and its subscales.

We established construct validity of the MBFES-A questionnaire by comparing the MBFES total and subscales’ scores of mothers who exclusively or partially breastfed their infants for one and three months to those who did not. We found that mothers who continued exclusive breastfeeding for one and/or three months had more overall satisfaction with breastfeeding, as well as more infant satisfaction/growth and maternal enjoyment/role attainment than those whose infants were shifted from breastfeeding to formula milk. These findings agree with those reported in the Japanese and Brazilian validation studies in which participants’ scores on the MBFES and its subscales were used for construct validity [24, 26]. In both studies, women who exclusively breastfed for one month had higher scores than those who were partially breastfeeding, and mothers who were not breastfeeding had the lowest scores. Furthermore, the Japanese study reported higher total MBFES scores and higher Maternal Satisfaction and Perceived Benefit to Baby subscales’ scores at four months in exclusively breastfeeding mothers, as compared to those who were not breastfeeding [24].

We also used maternal attitude towards breastfeeding, maternal breastfeeding knowledge, number of breastfed children, and the longest duration of previous breastfeeding as additional variables to test for construct validity. However, we found that maternal satisfaction with breastfeeding had modest positive correlations with maternal attitude towards breastfeeding (*r* = 0.30) and longer duration of previous exclusive breastfeeding (*r* = 0.27), and poor correlations with maternal breastfeeding knowledge and number of breastfed children (*r* = 0.12 for both). Interestingly, the association between the longest duration of previous exclusive breastfeeding and maternal satisfaction with the overall breastfeeding experience is comparable to the findings of previous MBFES validation studies, in which mothers who reported breastfeeding for longer than six weeks were more satisfied with their breastfeeding experience as compared to those who breastfed for shorter periods [15, 16, 26]. It is also noteworthy that the correlation coefficients of the associations between MBFES scores and the constructs used for construct validity in previous MBFES validation studies ranged between 0.25 and 0.39 [16, 24, 26], comparable to our correlation coefficients. Since these values are on the modest side, we recommend that future research validating this instrument in other settings use different constructs to establish a stronger construct validity.

Our study has strengths and limitations. It is the first study to provide a validated Arabic instrument that can measure perceived maternal satisfaction with the overall breastfeeding experience. It is interesting to note that whereas the Maternal Enjoyment/Role Attainment and Infant Satisfaction/Growth subscales were higher in mothers who were breastfeeding at one and three months, the Lifestyle/Body Image subscale scores were similar in all mothers, irrespective of their breastfeeding status. This is consistent with what has been reported in the Japanese MBFES validation study, in which this subscale was not associated with breastfeeding at one month [24], and in the revised Japanese MBFES where it was not associated with the intention to breastfeed [25]. Another strength of our study is its large sample size (*n* = 485), which exceeds the recommended seven to ten subjects per item of tool in instrument validation studies [31–33].

The main limitation of our study is its generalizability. Most of our participants are highly educated Lebanese women with middle or high income, and who live in an urban area, mostly from the capital city. Hence, they may not be representative of Lebanese women with lower education or income, or who live in non-urban areas of the country. Similarly, they may not be representative of other Arab women. These limitations call for further replication of our findings in other MENA countries, with special emphasis on new concepts to establish a stronger construct validity. Another limitation is the fact that the MBFES was administered by telephone survey instead of self-administration by the participants, as was originally done by leff, et al. [15] and Riordan, et al. [16]. However, since most of our participants are highly educated with university degrees, we believe that this limitation may have affected the instrument’s reliability to a minimal degree.

## Conclusions

The 26-item MBFES-A is a reliable and valid tool to assess maternal perceived overall satisfaction with breastfeeding. We found that women who continued breastfeeding for one and/or three months had higher total MBFES-A scores, as well as higher scores on the Maternal Enjoyment/Role Attainment and Infant Satisfaction/Growth subscales. The MBFES-A and its subscales are useful tools for investigators conducting breastfeeding research in the MENA countries that share the Arabic language. Further replication of our findings in other Arab contexts is needed.

## Supplementary Information

**Additional file 1.** Arabic 26-item MBFES.

## Data Availability

The datasets used and analyzed during the current study are available from the corresponding author on reasonable request.

## Abbreviations

BFK-A: Breastfeeding Knowledge Questionnaire-Arabic
EFA: Exploratory factor analysis
IIFAS-A: Iowa Infant Feeding Attitude Scale-Arabic
IQR: Interquartile range
KMO: Kaiser-Meyer-Olkin
MBFES: Maternal Breastfeeding Evaluation Scale
MBFES-A: Maternal Breastfeeding Evaluation Scale-Arabic
MENA: Middle East and North Africa
SD: Standard deviation
PCA: Principal component analysis
UNICEF: United Nations International Children’s Emergency Fund
WHO: World Health Organization

## References

[1] Victora CG, Bahl R, Barros AJD, Franҫa GVA, Horton S, Krasevec J (2016). Breastfeeding in the 21^st^ century: epidemiology, mechanisms, and lifelong effect. Lancet..

[2] Binns C, Lee M, Low WY (2016). The long-term public health benefits of breastfeeding. Asia Pac J Public Health.

[3] Victora CG, Horta BL, Loret de Mola CL, Quevedo L, Pinheiro RT, Gigante DP (2015). Association between breastfeeding and intelligence, educational attainment, and income at 30 years of age: a prospective birth cohort study from Brazil. Lancet Glob Health.

[4] Yan J, Liu L, Zhu Y, Huang G, Wang PP (2014). The association between breastfeeding and childhood obesity: a meta-analysis. BMC Public Health.

[5] Belfort MB, Rifas-Shiman SL, Kleinman KP, Guthrie LB, Bellinger DC, Taveras EM, Gillman MW, Oken E (2013). Infant feeding and childhood cognition at ages 3 and 7 years. Effects of breastfeeding duration and exclusivity. JAMA Pediatr.

[6] Bhutta ZA, Das JK, Rizvi A, Gaffey MF, Walker N, Horton S, Webb P, Lartey A, Black RE (2013). Evidence-based interventions for improvement of maternal and child nutrition: what can be done and at what cost?. Lancet..

[7] Wiklund PK, Xu L, Wang Q, Mikkola T, Lyytikäinen A, Völgyi E, Munukka E, Cheng SM, Alen M, Keinänen-Kiukaanniemi S, Cheng S (2012). Lactation is associated with greater maternal bone size and bone strength later in life. Osteoporos Int.

[8] Khasawneh W, Khasawneh AA (2017). Predictors and barriers to breastfeeding in north of Jordan: could we do better?. Int Breastfeed J.

[9] Al Juaid DA, Binns CW, Giglia RC (2014). Breastfeeding in Saudi Arabia: a review. Int Breastfeed J.

[10] El Shafei AM, Labib JR (2014). Determinants of exclusive breastfeeding and introduction of complementary foods in rural Egyptian communities. Global J Health Sci.

[11] Hendaus MA, Alhammadi AH, Khan S, Osman S, Hamad A (2018). Breastfeeding rates and barriers: a report from the state of Qatar. Int J Women's Health.

[12] Batal M, Boulghourjian C (2005). Breastfeeding initiation and duration in Lebanon: are the hospitals “mother friendly”?. J Pediatr Nurs.

[13] Chehab RF, Nasreddine L, Zgheib R, Forman MR (2020). Exclusive breastfeeding during the 40-day rest period and at six months in Lebanon: a cross-sectional study. Int Breastfeed J.

[14] United Nations International Children’s Emergency Fund. The state of the world’s children 2015: Executive summary. Reimagine the future Innovation for every child 2015. Available from: https://reliefweb.int/sites/reliefweb.int/files/resources/SOWC_2015_Summary_and_Tables_1.pdf. Accessed 17 March 2021.

[15] Leff EW, Jefferis SC, Gagne MP (1994). The development of the maternal breastfeeding evaluation scale. J Hum Lact.

[16] Riordan JM, Woodley G, Heaton K (1994). Testing validity and reliability of an instrument which measures maternal evaluation of breastfeeding. J Hum Lact.

[17] Schlomer JA, Kemmerer J, Twiss JJ (1999). Evaluating the association of two breastfeeding assessment tools with breastfeeding problems and breastfeeding satisfaction. J Hum Lact.

[18] Dennis CL, Hodnett E, Gallop R, Chalmers B (2002). The effect of peer support on breast-feeding duration among primiparous women: a randomized controlled trial. CMAJ..

[19] Semenic S, Loiselle C, Gottlieb L (2008). Predictors of the duration of exclusive breastfeeding among first-time mothers. Res Nurs Health.

[20] Cooke M, Sheehan A, Schmied VA (2003). Description of the relationship between breastfeeding experiences, breastfeeding satisfaction, and weaning in the first 3 months after birth. J Hum Lact.

[21] Sheehan A (1999). A comparison of two methods of antenatal breast-feeding education. Midwifery..

[22] Cooke M, Schmied V, Sheehan A (2007). An exploration of the relation-ship between postnatal distress and maternal role attainment, breast feeding problems and breast feeding cessation in Australia. Midwifery..

[23] Hoddinott P, Britten J, Prescott GJ, Tappin D, Ludbrook A, Godden DJ (2009). Effectiveness of policy to provide breastfeeding groups (BIG) for pregnant and breastfeeding mothers in primary care: cluster randomized controlled trial. BMJ..

[24] Hongo H, Green J, Otsuka K, Jimba M (2013). Development and psychometric testing of the Japanese version of the maternal breastfeeding evaluation scale. J Hum Lact.

[25] Hongo H, Green J, Nanishi K, Jimba M (2017). Development of the revised Japanese maternal breastfeeding evaluation scale, short version. Asia Pac J Clin Nutr.

[26] de Senna AFK, Giugliani C, Lagoa JCA, Bizon AMB, Martins ACM, Oliveirac CAV (2020). Validation of a tool to evaluate women’s satisfaction with breastfeeding for the Brazilian population. J Pediat.

[27] Tamim H, Ghandour LA, Shamsedine L, Charafeddine L, Nasser F, Khalil Y, Nabulsi M (2016). Adaptation and validation of the Arabic version of the infant breastfeeding knowledge questionnaire among Lebanese women. J Hum Lact.

[28] Charafeddine L, Tamim H, Soubra M, de la Mora A, Nabulsi M (2016). For the research and advocacy breastfeeding team. Validation of the Arabic version of the Iowa infant feeding attitude scale among Lebanese women. J Hum Lact.

[29] World Health Organization. The World Health Organization’s infant feeding recommendation. 2002. Available from: https://www.who.int/nutrition/topics/infantfeeding_recommendation/en/. Accessed 17 March 2021.

[30] Dziuban CD, Shirkey EC (1974). When is a correlation matrix appropriate for factor analysis? Some decision rules. Psychol Bull.

[31] Terwee CB, Bot SD, de Boer MR, van der Windt DA, Knol DL, Dekker J (2007). Quality criteria were proposed for measurement properties of health status questionnaires. J Clin Epidemiol.

[32] World Health Organization. Process of translation and adaptation of instruments. 2015. Available from: http://www.who.int/substance_abuse/research_tools/translation/en/. Accessed 17 March 2021.

[33] Anthoine E, Moret L, Regnault A, Sébille V, Hardouin JB (2014). Sample size used to validate a scale: a review of publications on newly-developed patient reported outcomes measures. Health Qual Life Outcomes.

