# Supplementary material for: Validation of the Arabic Maternal Breastfeeding Evaluation Scale (MBFES-A) among Lebanese women
Source: Int Breastfeed J. 2021 Aug 21;16:60. doi: 10.1186/s13006-021-00409-w (PMC8379770; doi:10.1186/s13006-021-00409-w)
Supplement: Supplementary file 1 — Additional file 1. Arabic 26-item MBFES. [file 13006_2021_409_MOESM1_ESM.pdf]

مقياس تقييم الأم للرضاعة الطبيعية

إسم الأم:________________________________

التوجيهات: لكل من الإفادات التالية، الرجاء أن تحددي كم توافقين أو لا توافقين بإختيارك الجواب الأقرب ملائمة مع رأيك:

	لا أوافق بشدة	لا أوافق	لا رأي لدي	أوافق	أوافق بشدة
1. مع الرضاعة الطبيعية أحسست بالسعادة الداخلية					
2. وقت الرضاعة هو وقت خاص لي مع طفلي					
3. طفلي لا يهتم للرضاعة الطبيعية					
4. طفلي يحب الرضاعة الطبيعية					
5. كان عبء علي أن أكون مصدر الغذاء الرئيسي لطفلي					
6. أحسست بالقرب الشديد من طفلي عندماكنت أرضعه					
7. طفلي كان متلهفا" للرضاعة					
8. الرضاعة كانت مرهقة جسديا"					
9. كان من المهم لي أن أكون قادرة على الإرضاع					
10. خلال فترة الرضاعة كان نمو طفلي ممتاز					
11. أنا وطفلي عملنا سويا" لكي تكون الرضاعة سلسة					
12. الرضاعة كانت تجربة جيدة لي في الرعاية كأم					
13. خلال فترة الرضاعة كنت أشعر بأني مقيدة كل الوقت					
14. الرضاعة كانت تهدئ طفلي عندما ينزعج أو يبكي					
15. الرضاعة كانت تعلي من نفسيتي					
16. كنت راضية جدا" لأنه كان بإمكاني إنتاج الغذاء لطفلي					
17. الرضاعة جعلتني أشعر بأني أم جيدة					
18. لقد كنت أستمتع فعلا" بالرضاعة					
19. خلال الرضاعة كنت أقلق بشأن إسترجاع شكل جسمي					
20. الرضاعة جعلتني أشعر بثقة أكبر كأم					

21. زاد وزن طفلي بشكل جيد على حليبي					
22. الرضاعة جعلت طفلي يحس بالإطمئنان أكثر					
23. كان سهلا" علي أن أوفق بين رضاعة طفلي و نشاطاتي الأخرى					
24. الرضاعة جعلتني أشعر كالبقرة					
25. طفلي لم يكن مرتاحا" خلال الرضاعة					
26. الرضاعة كانت رائعة بالنسبة لي					
